# Pig‐specific RNA editing during early embryo development revealed by genome‐wide comparisons

**DOI:** 10.1002/2211-5463.12900

**Published:** 2020-06-25

**Authors:** Tongtong Li, Qun Li, Hao Li, Xia Xiao, Dawood Ahmad Warraich, Ning Zhang, Ziyun Chen, Junyao Hou, Tong Liu, Xiaogang Weng, Zhonghua Liu, Jinlian Hua, Mingzhi Liao

**Affiliations:** ^1^ College of Life Sciences Northwest A&F University Yangling China; ^2^ Chinese Academy of Sciences Center for Excellence in Molecular Plant Sciences Shanghai Center for Plant Stress Biology Chinese Academy of Sciences Beijing China; ^3^ University of Chinese Academy of Sciences Beijing China; ^4^ Key Laboratory of Animal Cellular and Genetic Engineering of Heilongjiang Province College of Life Science Northeast Agricultural University Harbin China; ^5^ College of Veterinary Medicine Shaanxi Centre of Stem Cells Engineering & Technology Northwest A&F University Yangling China

**Keywords:** early embryo development, *ECI1*, *HNF1A*, *HOX* family, pig, RNA editing

## Abstract

Posttranscriptional modification of mRNA sequences through RNA editing can increase transcriptome and proteome diversity in eukaryotes. Studies of fetal and adult tissues showed that adenosine‐to‐inosine RNA editing plays a crucial role in early human development, but there is a lack of global understanding of dynamic RNA editing during mammalian early embryonic development. Therefore, here we used RNA sequencing data from human, pig and mouse during early embryonic development to detect edited genes that may regulate stem cell pluripotency. We observed that although most of the RNA editing sites are located in intergenic, intron and UTR, a few editing sites are in coding regions and may result in nonsynonymous amino acid changes. Some editing sites are predicted to change the structure of a protein. We also report that *HNF1A*, *TBX3*, *ACLY*, *ECI1* and *ERDR1* are related to embryonic development and cell division.

## Graphical abstract

RNA sequence data from early embryonic development in human, mouse, and pig were used to detect edited genes that may regulate stem cell pluripotency. Non‐synonymous editing has lower frequency than synonymous editing. Humans and pigs share more editing genes during embryonic development. Edited *HNF1A* can change its protein secondary and tertiary structures while *ECI1* only changed the secondary structure.

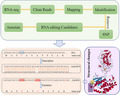

**Abbreviations**
ADAR: adenosine deaminases acting on RNA
A‐to‐I: adenosine‐to‐inosine
CDS: coding region
Ess: Essential
GEO: Gene Expression Omnibus
GIC: Gene Importance Calculator
GO: Gene Ontology
Noness: nonessential
RNA‐seq: RNA sequencing
SNP: single‐nucleotide polymorphism

Embryonic stem cells have totipotency and can develop into various tissues to form a complete embryo [1]. Considering their long‐term growth capacity of maintaining normal karyotype and pluripotency, they are important materials for studying early developmental, cell differentiation, drug discovery and future regenerative medicine. Although there are many related kinds of research, the molecular regulation mechanism about the development of early embryo in mammals is still unclear.

RNA editing is the process of altering genetic information at the mRNA level. Specifically, it will present in the transcription of genes. Due to deletion, insertion or substitution of a nucleotide [2, 3], the sequence of gene transcript will not be complementary to its coding sequence in DNA, and the amino acid composition of the protein produced by the translation will be different from the sequence of the gene. RNA editing can occur in both coding regions (CDSs) and non‐CDSs. Most RNA editing is observed at the first or second position of the genetic codon [4], thereby directly altering the encoded amino acid. In particular, RNA editing can generate start or stop codons or remove stop codons to produce different sizes of proteins. As an important regulatory mechanism of transcription modification, RNA editing plays an important role in the realization of gene function [5, 6, 7] and the diversification of gene products [8, 9, 10].

Currently, studies have shown that RNA editing is associated with a variety of human cancers and contributes to the production and maintenance of cancer stem cells that control cancer progression and drug resistance [11]. In the study of sexual reproduction of fungi, adenosine‐to‐inosine (A‐to‐I) editing showed stage specificity [12]. In addition, with the development of next‐generation sequencing technology, a wide range of mRNA modifications and their effects on mammals have been discovered [6, 13, 14]. There is also evidence that RNA modification plays an important role in stem cells [15]. Studies of fetal and adult tissues showed that A‐to‐I RNA editing plays a crucial role in early human development [16], but systematic dynamic analysis is still needed for RNA editing during the development of an early embryo in mammal.

In this work, we studied the dynamic process of RNA editing based on RNA sequencing (RNA‐seq) datasets about early embryonic development. The cell types of early embryonic development include two‐cell, four‐cell, eight‐cell, morula and blastocyst. Three species were analyzed in our work, including human, mouse and pig. RNA editing occurs throughout the early embryonic development process, and the highest number of editing sites is generally in the eight‐cell stage. Interesting, although the majority of editing sites are in non‐CDSs, a few nonsynonymous edited genes occur. Overall, this study comprehensively analyzed and compared the RNA editing of early embryonic development in human, mouse and pig. Finally, some edited genes related to stem cell pluripotency, such as *HNF1A*, *ECI1* and *HOX* families, were found.

## Materials and methods

### Transcriptome data and read mapping

We obtained RNA‐seq data of whole early embryonic stages from Gene Expression Omnibus (GEO; https://www.ncbi.nlm.nih.gov/geo/) (Table S1). The RNA‐seq from pig has been uploaded to the GEO database with GSE139512. Samples were collected from pig preimplantation embryos. RNA was harvested using TRIzol reagent. Illumina TruSeq RNA Sample Prep Kit (Catalog #FC‐122‐1001) was used with 1 µg total RNA for the construction of sequencing libraries. RNA libraries were prepared for sequencing using standard Illumina protocols. Smart‐Seq2 method was used to amplify the single‐cell sample. The sample cDNA was fragmented into 300 bp; then we performed terminal repair, addition of A, and ligation of sequencing adapters, and amplified. The PCR amplification product with 350–450 bp was extracted. RNA sequences of human and mouse used to identify editing sites were generated for this study and obtained from Liu W *et al.* [17] Wu J *et al.* [18], Yan L *et al.* [19] and Fan X *et al.* [20]. RNA libraries were sequenced on Illumina platform using single cells. Yan *et al.* sequenced the libraries as single‐end reads [19], whereas others obtained pair‐end reads. Reads length of the data we downloaded from GEO was 100 bp and of the pig data was 150 bp. Most of the molecules extracted from the data we used was total RNA, and we integrated RNA‐seq at the same stage to improve the sequencing depth and coverage. All reads were trimmed and then aligned to the genome. Use exonerate v2.4.0 [21] was used to reduce misalignment caused by reads to the location of multiple sites and misalignments around splice‐site junctions. We used Trim Galore (https://github.com/FelixKrueger/TrimGalore/releases) to filter out reads from all studies that contained adapters and low quality using ‘‐‐quality 33 ‐‐phred33 ‐‐stringency 3 ‐‐length 20 –paired’. Reads were aligned to the human genome (hg38), the pig genome (susScr11) and the mouse genome (mm10) with HISAT2 (http://github.com/infphilo/hisat2) [21], respectively. The ensemble gene set is used to generate the transcriptome index. The files were converted to the bam format available in SAMtools 1.16 [22]. We tried to remove duplicates of these files by Picard tools (http://broadinstitute.github.io/picard/), and clean reads were integrated. Candidate RNA editing sites were detected by REDItools [23].

### Identification of RNA editing candidates

We used REDItools to detect candidate sites. Finally, we collected all mismatches between the earlier reads and the reference genome. Multiple test correction was performed to correct the *P*‐value using the p.adjust function of r program, and editing sites with a false discovery rate >0.05 were removed. The bcftools [24] was used to integrate the single‐nucleotide polymorphism (SNP) location information in the dbSNP database (http://www.ncbi.nlm.nih.gov/SNP/), Ensembl (ftp://ftp.ensembl.org/pub/release‐98/variation/) and Sanger Institute (ftp://ftp‐mouse.sanger.ac.uk/REL‐1807‐SNPs_Indels/mgp.v6.merged.norm.snp.indels.sfiltered.vcf.gz). Then all SNPs in the databases were removed with bedtools [25]. For rare SNPs filtering, as Ramaswami points out, true editing sites often exist in different individuals, and rare SNPs are probably not present [17]. So we also filtered out candidate RNA variants if their editing frequency = 1. These methods are used to reduce the interference of gene mutations. We downloaded the repeat sequences from the University of California, Santa Cruz (http://genome.ucsc.edu). Alu sequences were extracted with the Python program; then the editing sites on the Alu were analyzed.

### Annotate RNA editing sites

We used the annovar software [27] to annotate RNA editing. The referential gene set used for annotation was from Ensembl and National Center for Biotechnology Information databases. The annovar was written by the Perl programming language, and it classifies the RNA editing sites we have obtained to determine which positions of the genes are causing protein mutations. Finally, the results showed that there were not many mutations in the exon.

### Essential and nonessential genes

Essential (Ess) and nonessential (Noness) genes from human and mouse were obtained from the OGEE (Online GEne Essentiality) database. The human gene in the database contained datasets from multiple laboratories and was processed by different experiments. Therefore, this may misclassify many human Ess genes as Noness genes and mismatch some Noness human genes into Ess genes. There is only one dataset of mice in the database, resulting in insufficient data. So we used Gene Importance Calculator (GIC) [26] to calculate the importance of the editing genes. So there were two types of genes, Ess genes and Noness genes. On this basis, we still found there were significant differences of edited gene numbers between Ess and Noness genes. All of the analysis of the χ^2^ test and *F* test for the earlier data was based on r program.

### Gene Ontology analysis

The gene set enrichment analysis was performed on r package clusterProfiler [28] and Metascape (http://metascape.org) [29]. To identify Gene Ontology (GO) categories that are enriched in a specific set of genes, we performed GO analysis not only on all the genes that have RNA editing but also on genes that have undergone nonsynonymous editing.

### Protein structure prediction

We downloaded the gene sequence from National Center for Biotechnology Information and modified the editing site in the sequence. PSIPRED was used to predict the secondary structure of the two sequences [30]. We retain the genes for the amino acid changes caused by RNA editing. Meanwhile, we used SWISS‐MODEL to predict the tertiary structure of proteins [31], and we also used Tm‐align [32] to ensure that the structure views are intercomparable. Discovery Studio (https://www.3dsbiovia.com/products/collaborative‐science/biovia‐discovery‐studio/) was used to visualize structures.

## Results

### Editing sites vary in different stages and chromosomes

**Fig. 1:**
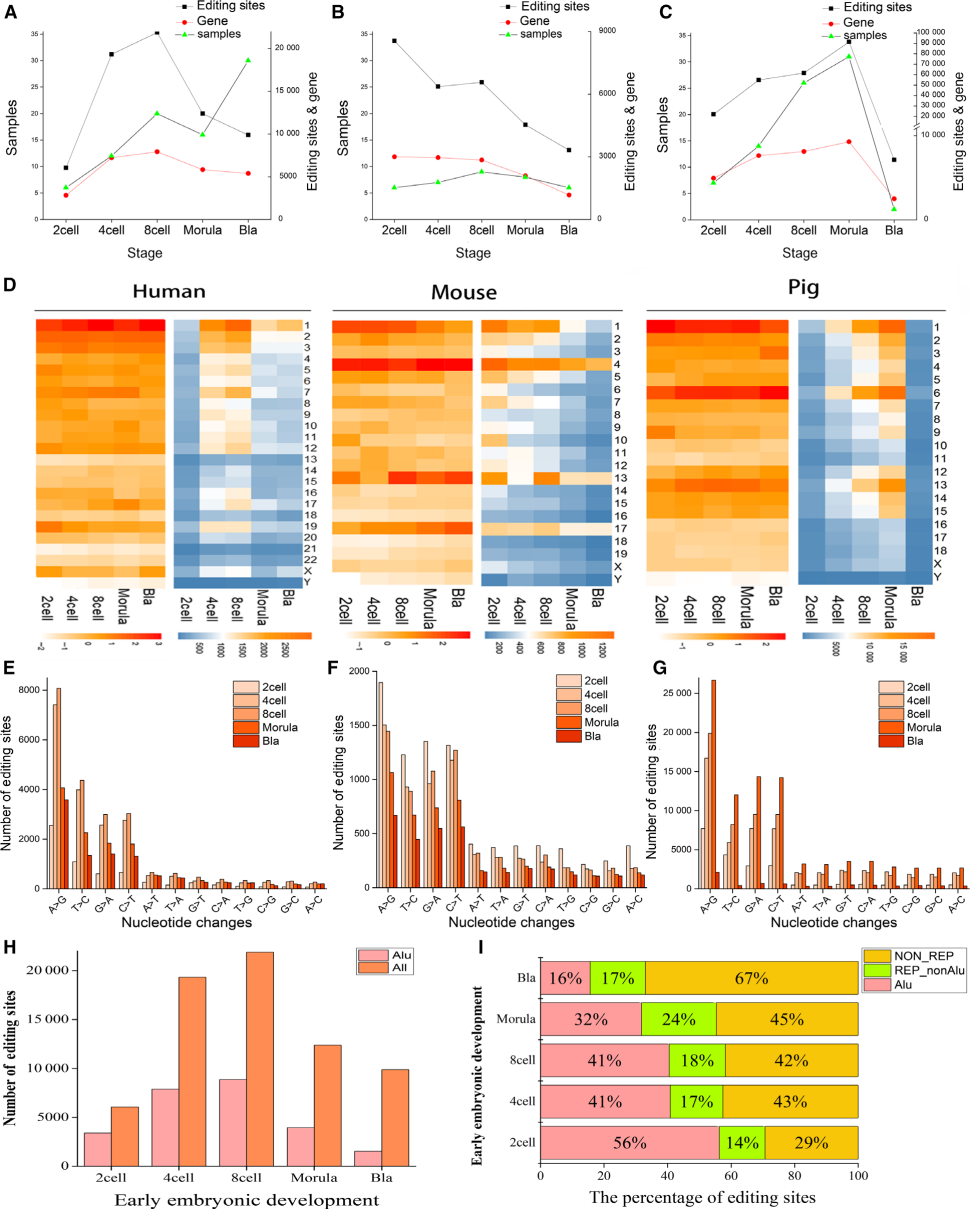
Distributions of RNA editing in different species, stages and chromosomes. (A–C) The relationships between the number of samples, the number of editing sites and the number of edited genes in different cells. The *x* axis is the phase of early embryonic development, and the *y* axis is the number of samples (left), the number of editing sites and the number of edited genes after deduplication (right). (D) The distribution of editing site numbers among different chromosomes. (E–G) Bar graphs represent nucleotide changes found by the REDItools. The *x* axis is 12‐nucleotide changes, and the *y* axis is the number of edits. From left to right are human, mouse and pig. (H) Distribution of the Alu region and the number of editing sites during early embryonic development. Red represents the number of editing sites occurring in the Alu region, and orange represents the number of all RNA editing sites. (I) The percentage of editing sites in the repeat sequence for human. The *x* axis is the percentage of editing sites in the Alu, and the *y* axis is the different developmental stages. Red is the proportion of the editing sites occurring in the Alu region, green is the repeating region except Alu, and yellow is the non‐repeating region.

We analyzed five stages in the early embryonic development of human, mouse and pig, including two‐cell, four‐cell, eight‐cell, morula and blastocyst (Table S1). We can find that when the number of samples increases, the possibility of RNA editing detection will increase through Fig. 1A–C. It is also found that RNA editing differs in different stages, chromosomes and editing types. In human, RNA editing sites are concentrated on chromosome 1. Editing sites in pig are not only concentrated on chromosome 1 but also on chromosome 6. Unlike human and pig, RNA editing occurs mostly on chromosomes 1, 4, 13 and 17 in mouse (Fig. 1D). We then focused on the chromosomes that had a large number of edits and made function enrichment analysis. Results showed that the edited genes on chromosome 4 in mouse mainly related to RNA processing (Fig. S1), especially related to pre‐miRNAs, and RNA editing genes in chromosome 17 in mouse are involved in immune processes such as antigens (Fig. S1). Moreover, the enrichment of chromosome 6 in pig was mainly related to lipid metabolism (Fig. S1). The main types of nucleotide substitutions in human are A‐to‐I and uridine‐to‐cytidine (U‐to‐C), whereas in pig and mouse, cytidine‐to‐uridine (C‐to‐U) and guanosine‐to‐adenosine (G‐to‐A) are also in the top of the list (Fig. 1E–G). False‐positive results were reduced by using exonerate v2.4.0 (Fig. S2). A‐to‐I substitution is more common in human, mainly due to the presence of Alu repeats in its genome. In the early development of human embryos, 25 686 A‐to‐I RNA editing events were identified (Table 1). A‐to‐I events occurred mainly in the Alu region (Table S2 and Fig. S2). The Alu sequence is a moderately repeating sequence unique to primates [33, 34]. In addition, the number of RNA editing events in the Alu regions at the eight‐cell stage remains the highest (Fig. 1H). However, the proportion of editing events in the Alu region is reduced during the development of the early embryo (Fig. 1I and Table S3). The results are consistent with previous research. Shtrichman *et al*. found that the RNA editing activity of Alu in human fetal tissue samples decreased relative to adult tissue samples [16]. The reason is not clear, and it may be related to the need for division and differentiation during embryonic development.

**Table 1:** Numbers and percentages of different types of RNA editing in human.

Region	Total	A→C	A→I	A→U	C→A	C→G	C→U	G→A	G→C	G→U	U→A	U→C	U→G
Number of editing sites
Two‐cell	6059	70	2551	264	162	78	665	605	71	245	155	1087	106
Four‐cell	19 336	208	7411	533	246	264	2764	2570	285	320	507	3985	243
Eight‐cell	21 882	271	8073	663	393	334	3036	2995	312	471	624	4370	340
Morula	12 397	197	4068	555	271	186	1807	1843	198	332	447	2261	232
Blastocyst	9882	208	3583	527	246	130	1320	1406	169	267	436	1346	244
All	69 556	954	25 686	2542	1318	992	9592	9419	1035	1635	2169	13 049	1165
Percentage of editing sites
Two‐cell	100.00	1.16	42.11	4.36	2.67	1.29	10.97	9.98	1.17	4.04	2.56	17.94	1.75
Four‐cell	100.00	1.08	38.33	2.76	1.27	1.37	14.29	13.29	1.47	1.65	2.62	20.61	1.26
Eight‐cell	100.00	1.24	36.90	3.03	1.80	1.53	13.87	13.69	1.43	2.15	2.85	19.97	1.55
Morula	100.00	1.59	32.81	4.48	2.19	1.50	14.58	14.87	1.60	2.68	3.61	18.24	1.87
Blastocyst	100.00	2.10	36.27	5.33	2.49	1.32	13.35	14.23	1.71	2.70	4.41	13.62	2.47
All	100.00	1.37	36.93	3.65	1.89	1.43	13.79	13.54	1.49	2.35	3.12	18.76	1.67

### Nonsynonymous RNA editing in the early embryo

**Fig. 2:**
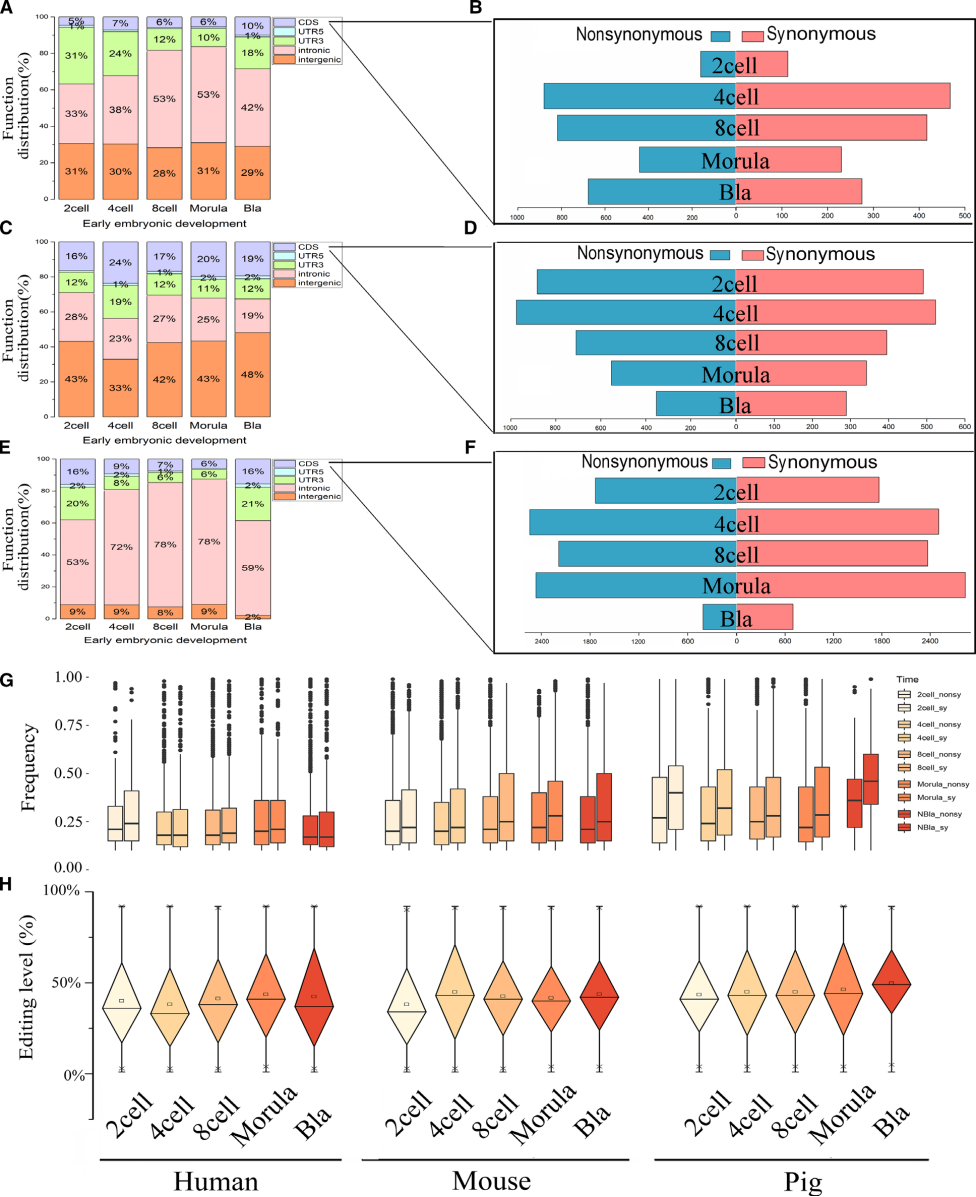
Distribution of RNA editing functional regions and editing level trends during early embryo development. (A, C, E) Function distribution of RNA editing sites. From top to bottom are human, mouse and pig. (B, D, F) The number of nonsynonymous and synonymous edits in CDSs. The number of nonsynonymous edits is in blue, and the number of synonymous edits is in red. (G) Nonsynonymous and synonymous editing frequency in different cells. (H) Editing levels during early embryo development. The *x* axis is the different developmental stages. From left to right are human, mouse and pig. Red is the proportion of the editing sites occurring in the Alu region, green is the repeating region except Alu and yellow is the nonrepeating region.

According to our analysis, RNA editing in early embryo occurs mainly in intergenic regions, introns and UTRs, regardless of species (Fig. 2A,C,E). At the same time, we identified 4159, 4516 and 16 202 editing sites in protein CDSs of human, mouse and pig, respectively. In human, 2783 editing sites that result from nonsynonymous changes were detected, and they occupied about two‐thirds of the total RNA editing number (Fig. 2B). Similar results were found in mouse and pig, with 2923 and 8196 nonsynonymous RNA editing sites, respectively (Fig. 2D,F). These results indicate that RNA editing may play important roles during early embryo development.

To explore the relationship of RNA editing level in early embryonic development, we observed the distribution in the three species. RNA editing level is the ratio of the number of RNA‐seq reads, which is different from the reference base by the total number of reads covering the site [35]. We reserved the editing sites with the edit frequency in the range of greater than 0 and less than 1, and the result showed that the median of editing level remained between 45% and 55% (Fig. 2H). Similar results have also been observed in the study of Qiu *et al*. [36]. They believe that this is due to the difference in the expression levels of *ADAR1*, *ADAR2* and *ADAR3* genes at different stages [36]. Therefore, we speculate that this tendency is related to the expression level of RNA editing enzymes during early human embryonic development. We noticed the increase of RNA editing level occurred in the blastocyst stage in pig, indicating that the editing enzyme will be significantly high expression in this period. Interesting, we also found that not only synonymous mutation but also nonsynonymous mutation pig has higher editing levels in CDSs than human and mouse (Fig. 2G,H). However, human has the lowest frequency of editing in CDSs.

### RNA editing trends to present in nonessential genes

**Fig. 3:**
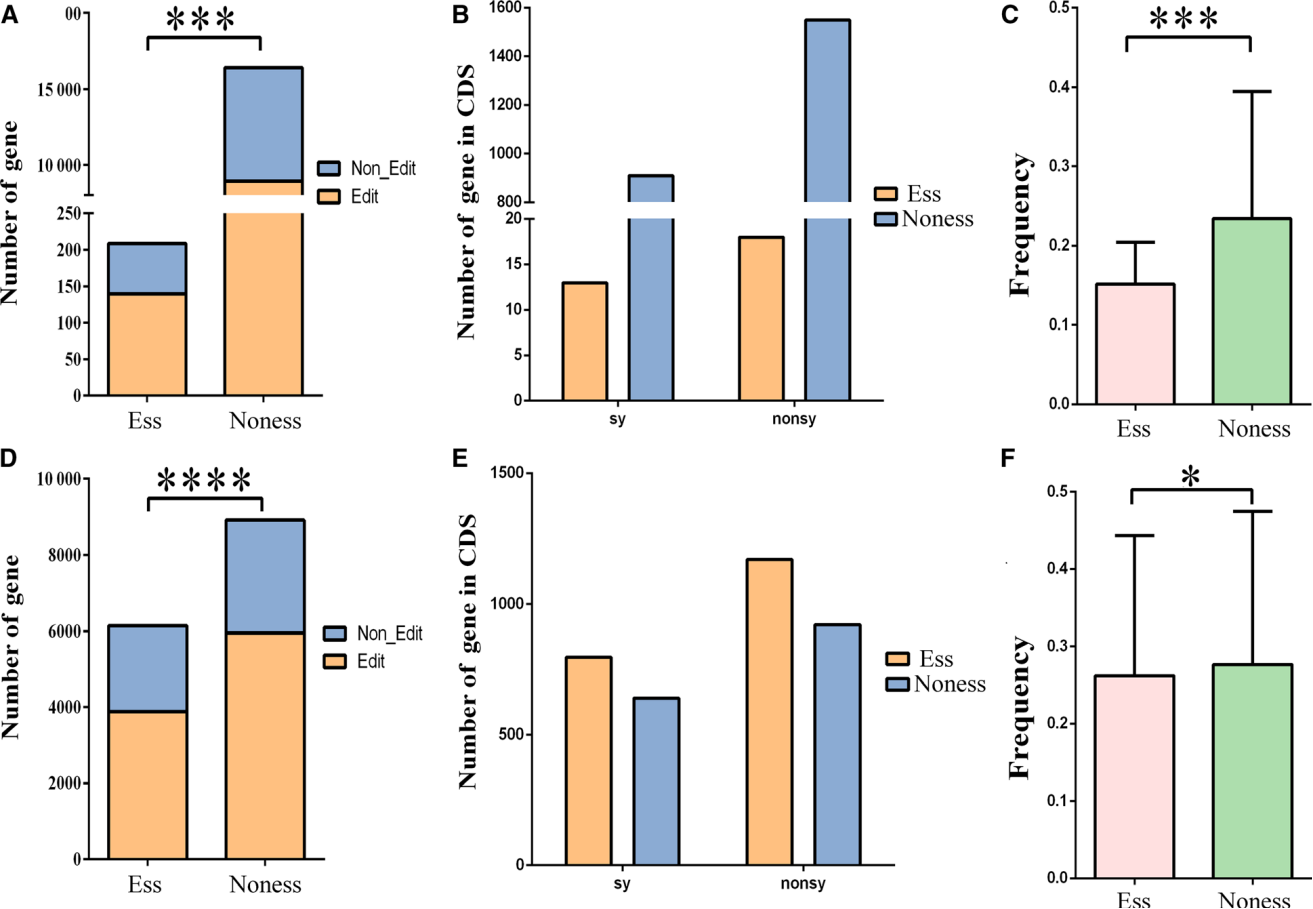
RNA editing sites in Ess and Noness genes. (A, D) Differences in the number of Ess genes between edited and unedited genes (*P* = 0.0003 in A; *P* < 0.0001 in D). The unedited genes here refer to the genes we obtained from OGEE and GIC, which are not present in the edited gene sets we have detected. (B, E) Edited gene numbers in CDSs are different between Ess and Noness genes (*P* = 0.2851 in B;* P* = 0.3795 in E). (C, F) The editing level in Ess genes is lower than in Noness genes. The results of human (A–C) and of mouse (D–F) are shown. The *P* values are from χ^2^ tests (A, B, D, E) and from *F* test (C, F). Ess, Essential; Noness, Non‐essential; nonsy, predicted nonsynonymous substitution; sy, predicted synonymous substitution.

To investigate whether the editing site is biased in the gene, we identified the necessity of the editing gene. Ess genes are those that are vital to the survival of organisms, whereas the rest are not necessary. They depend on external conditions, not intrinsic properties. First, we obtained Ess and Noness genes of human and mouse from the OGEE database [37]. Then, to reduce the impact of multiple datasets in human, we obtained 14 571 genes after removing the repetitive and controversial genes. There was only one dataset in mouse, including 9042 genes (Table S4). We also used GIC to calculate the importance of 3776 editing genes in mouse. Genes with GIC score >0.5 were recorded as Ess genes. Thus, the number of Ess genes in mouse increased to 1705, and the number of Noness genes increased by 2072 (Table S4). In humans, we found 140 Ess genes for RNA editing and 8934 Noness genes. Specific data are stored in Table S4. Among them, the number distribution of the edited gene number on the Ess gene and the Noness gene was significantly different from that of the unedited gene (*P* = 0.0003, Fig. 3A). Similarly, results were observed in mouse (*P* < 0.0001, Fig. 3D). Then, we counted the Ess and Noness genes at the editing site of the CDS (Fig. 3B,E). Also, in CDSs, the number of nonsynonymous edits in human is significantly higher than synonymous editing (Fig. 3B), and the same result in mouse (Fig. 3E). To investigate whether these nonsynonymous editing genes differ in the necessity of gene, we first divided these nonsynonymous editing genes into two categories according to necessity, and then performed *F* test on their editing frequency. Strikingly, there was a significant difference in the editing frequency of Ess and Noness genes (*P* = 0.0010 in Fig. 3C and *P* = 0.0335 in Fig. 3F). This observation is consistent with the results in Fig. 2G, where nonsynonymous editing has a lower frequency than synonymous editing. This is consistent with previous studies. In summary, we verified that RNA editing tends to present in Noness genes, and that similar results exist in human and mouse.

### Function of specific nonsynonymously RNA edited genes

**Fig. 4:**
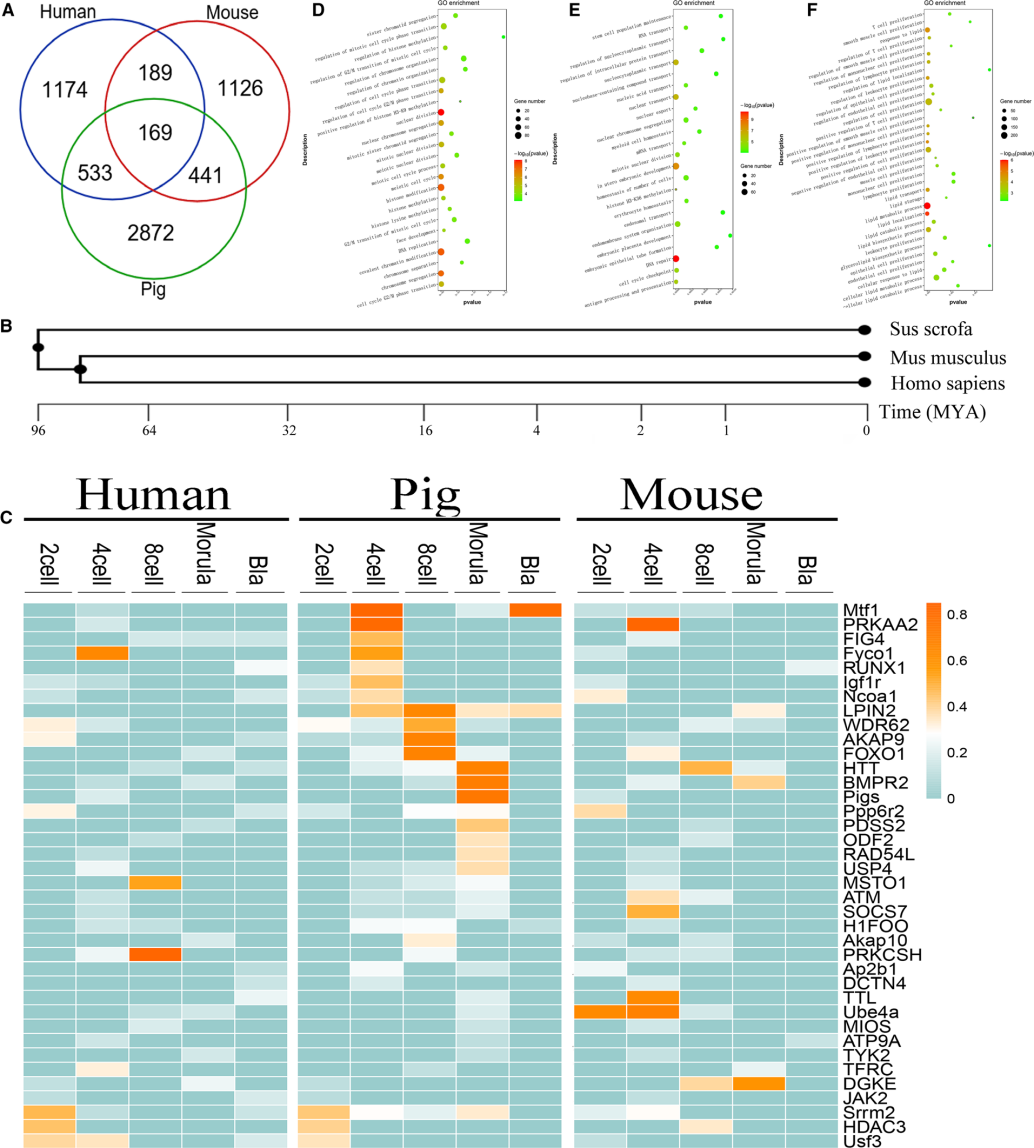
The intersection of nonsynonymous editing genes in three species. (A) The Venn diagram represents the intersection of all nonsynonymous editing genes in the three species. (B) Tree diagram of the origin of three species. (C) The editing frequency of some intersection genes of human, pig and mouse in different cells. It highlights some of the genes associated with pigs. (D–F) GO enrichment analysis of all nonsynonymous editing genes. From left to right are human, mouse and pig.

We combined the earlier genes, including 2065 genes in human, 1925 in mouse and 4015 in pig (Fig. 4A). Based on all of the numbers of nonsynonymous editing genes, we found that they present a U‐shaped trend during early embryonic development (Fig. S3). According to the selected genes, we know that there are more intersections between human and pig edited genes, and the relationship is closer (Fig. 4A). The tree diagram shows a close relationship between human and mouse (http://www.timetree.org/) [39] (Fig. 4B). The close relationship between human and mouse is based on the whole‐genome sequencing, according to past research. We found that human and pig shared more genes during embryonic development. The percentage of RNA editing genes in both human and pig accounts for 10.8% of all nonsynonymous editing genes (Fig. 4A). All three species had 169 editing genes, accounting for 2.6%. Enrichment analysis of these 169 genes showed that they were mainly related to signal regulation, metabolic processes and reproductive system development (Fig. S4 and Table S5). Differential RNA editing levels were observed within 169 genes (Fig. 4C and S5). The editing level of these genes is dynamic, high or low, especially in the case of incomplete genome annotation in pigs, among which there may be undeleted SNPs.

GO analysis indicates that these genes can be divided into genes that play a role in basic processes (energy production, DNA repair, RNA processing, mitosis) and are involved in developmental differentiation (cell differentiation, regulation of cell proliferation, embryonic organ development, signal regulation and histone methylation) (Fig. 4D–F and Tables 2 and S6). In particular, GO analysis of pig edited genes showed that they are mostly related to metabolic processes, especially lipid metabolism (Fig. 4F). One reason is that embryonic development requires a variety of energy support, and the other reason is based on the research of Zhang *et al*. [39] and Suzuki *et al*. [40], in which a large number of lipids are present in early embryos and have a positive effect on their development.

**Table 2:** GO analysis for genes that produced nonsynonymous editing. Only significant GO terms with an enrichment value *P* < 0.005 are presented.

Species	GO_term	Example gene	*P*‐value	No. of genes
Human	Regulation of chromosome organization	*APC/RIF1/JARID2*	0.000286512	3
	Regulation of histone methylation	*RIF1/JARID2*	0.000787868	2
	Maintenance of cell number	*TBX3/RIF1/POU5F1*	0.001130463	3
	Embryonic germ layer development	*INHBA/DVL1/POU5F1/BMPR2/ACVR2A/AXIN1*	4.89E−8	6
	Regulation of signaling pathway	*APC/DVL1/FZD6/AXIN1*	3.62E−5	4
	Stem cell population maintenance	*TBX3/RIF1/POU5F1*	0.00079208	3
	Regulation of stem cell proliferation	*TBX3/PAX6*	8.80E−5	2
Pig	Lipid metabolic process	*ACLY/CLN3/AGK/PTGR1/FAR1/PNPLA4/HADHA*	4.52E−5	67
	Cellular lipid metabolic process	*CLN3/AGK/PTGR1/PNPLA4/HADHA*	0.00054275	48
	Alcohol metabolic process	*PMVK/HMGCR/LDLR/CYP7A1/INSIG2/PRKAA2*	0.000344265	21
	Organic acid catabolic process	*HYAL2/HADHA/DLST/ETFB/GOT1/AMT*	0.000512426	16
	Cell development and differentiation	*PRKAR2A/FZR1/PRKAR1A/GDF9/PDE3A*	4.17E−6	12
	Ion homeostasis	*CLN3/NUBP1/SFXN1/ATP1A1/SLC9A1*	0.000277434	37
	Multiorganism reproductive process	*PRKAR2A/FZR1/PRKAR1A/GDF9/CD46*	0.00055712	43
	Regulation of cell maturation	*PRKAR2A/FZR1/PRKAR1A/GDF9/CDC20*	3.15E−5	11
Mouse	DNA repair	*Usp1/Ticrr/Smc4/Rbbp8/Uhrf1/Pnp*	3.6632436754777E−11	35
	RNA transport	*Tpr/Alkbh5/Nup214/Xpo5/Seh1L/Ahctf1*	0.000177271	15
	Interkinetic nuclear migration	*Tacc3/Hhex/Hook3/Dock7*	2.83E−583374166	4
	*In utero* embryonic development	*Ttn/Cnot1/Acvr1/E2f8/Tbx3/Hectd1/Chd7*	0.001310038	7
	Multicellular organism growth	*Celf1/Chd7/Rmi1/H2‐Q2/Daxx*	0.00216228	20
	Antigen processing	*H2‐Dmb2/H2‐Dmb1/H2‐Dma/H2‐Q1/H2‐Q2*	3.01E−6	18

### RNA editing can change the secondary or tertiary structure of proteins

**Fig. 5:**
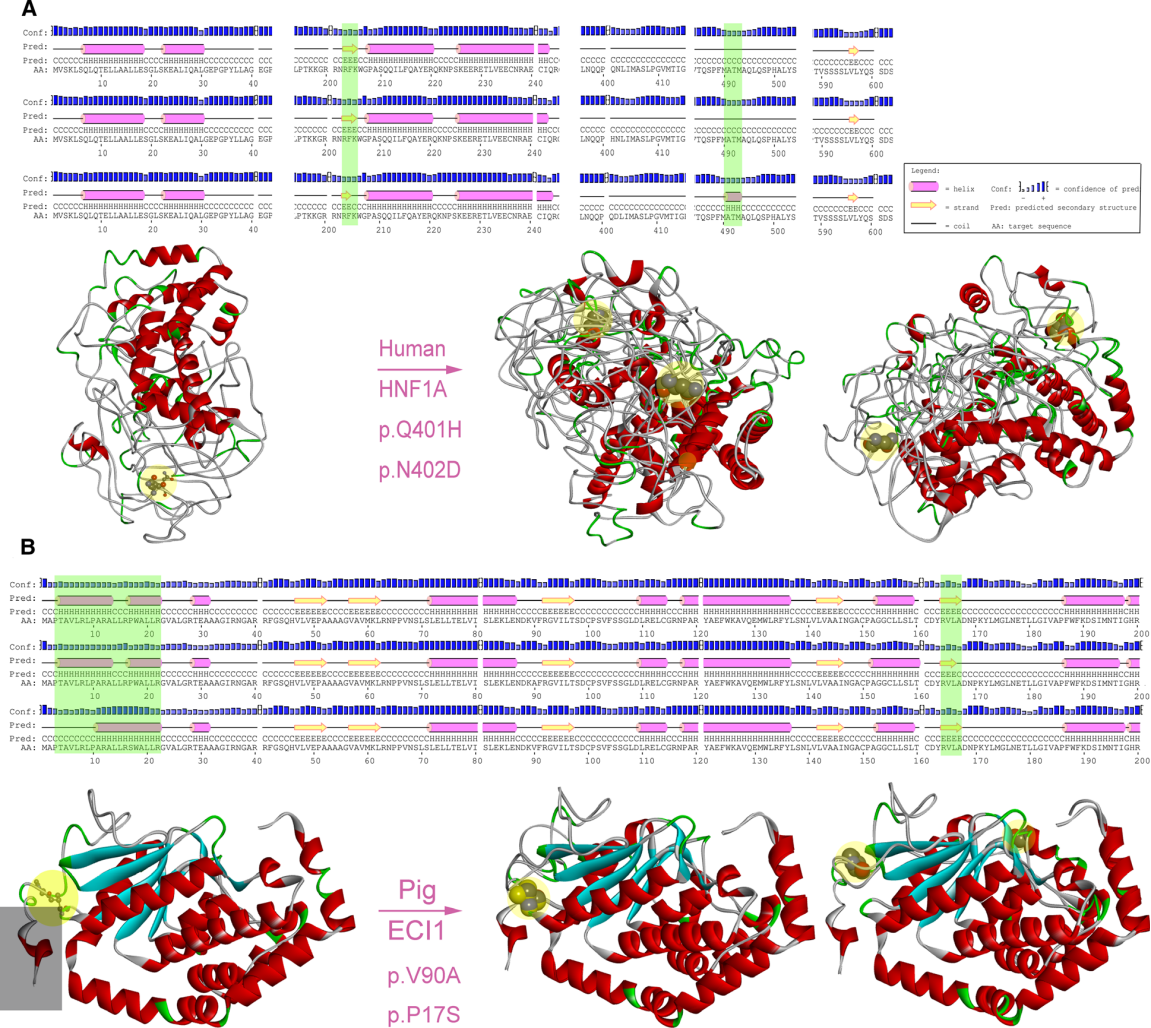
Protein structure prediction. (A) Structure prediction of *HNF1A* gene protein. The editing sites at 401 did not change the secondary structure of the protein. (B) In the case of *ECI1* in pigs, the editing sites changed the secondary structure of the protein and affected the tertiary structure. Yellow circles: amino acids before and after editing; gray rectangle: changed tertiary structure. Left: structure without RNA editing (a chain); right: the structure before and after RNA editing (two chains) showed the superposed full‐atom structure of the entire chain. A, alanine; D, aspartic acid; H, histidine; N, asparagine; P, proline; Q, glutamine; S, serine; V, valine.

Finally, we selected representative genes, such as the human *HOX* family genes and *RIF1*; the pig genes included *CD46*, *ACLY*, *CLN3*, among others; and the mouse genes were *H2‐DMA*, *KHDC1B*, *HJURP*, *ERDR1* and so on. The functions are mainly related to the regulation, proliferation and development of embryonic stem cells and energy metabolism (Table 3). Through predicting the protein structure of the earlier genes, the result shows that some have changed the secondary structure and others have changed the tertiary structure. For example, edited *ECI1* and *HNF1A* can change its protein secondary and tertiary structures (Fig. 5). Moreover, before the RNA editing of *ECI1*, the amino acids at positions 17 and 90 were close to each other, and when they were edited, they separated, resulting in a tertiary structural change (Fig. S6). *HNF1A*‐related long noncoding RNAs (lncRNAs) are known to be involved with the regulation of proliferation and migration of esophageal adenocarcinoma cells [41]. And Zhang *et al*. [42] study found that *ECI1* is related to lipid metabolism. Among these proteins, *ECI1* is an auxiliary enzyme in the beta‐oxidation of polyunsaturated fatty acids.

**Table 3:** Genes related to embryonic stem cell characteristics were selected based on GO analysis.

Species	Gene	Chromosome	Functional description
Human	*RIF1*	chr2	Transcriptional regulatory network in embryonic stem cell and signaling pathways regulating pluripotency of stem cells
	*POU5F1(Hox)*	chr6	This gene encodes a transcription factor containing a POU homeodomain that plays a key role in embryonic development and stem cell pluripotency Transcriptional regulatory network in embryonic stem cell
	*ZFHX3(Hox)*	chr16	Transcriptional regulatory network in embryonic stem cell and circadian rhythm–related genes
	*HNF1A(Hox)*	chr12	ERK signaling and adipogenesis
	*MEIS1(Hox)*	chr2	Homeobox genes, of which the most well‐characterized category is represented by the *HOX* genes, play a crucial role in normal development Transcriptional regulatory network in embryonic stem cell
Pig	*CD46*	chr9	The protein encoded by this gene may be involved in the fusion of the spermatozoa with the oocyte during fertilization Complement and coagulation cascades and complement pathway
	*ACLY*	chr12	ATP citrate lyase is involved in lipogenesis and cholesterol production Metabolism and integration of energy metabolism
	*CLN3*	chr3	This gene encodes a protein that is involved in lysosomal function Cell migration and transport; metabolic process
Mouse	*H2‐DMA*	chr17	Major histocompatibility complex class II protein complex binding Multicellular organism development; cell development process; development of the immune system
	*KHDC1B*	chr1	Activation of cysteine‐type endopeptidase activity involved in apoptotic process Apoptotic process; biological regulation
	*HJURP*	chr1	Chromosome segregation Cell cycle, mitotic and chromosome maintenance
	*ERDR1*	chrY	Negative regulation of cell migration, cell proliferation; somatic stem cell population maintenance

ERK, Extracellular signal‐regulated kinase.

## Discussion

RNA samples were extracted from early embryonic development tissues to detect RNA editing events. We found that RNA editing does exist in early human embryogenesis. After obtaining the candidate genes, we not only compared the editing events of five embryonic stages but also analyzed the differences of RNA editing among human, mouse and pig. Finally, we selected candidate edited genes that are related to the characteristics of stem cells.

Our study found that the number of RNA editing sites has little to do with the number of samples and is related to physiological needs during embryonic development. We found that the number of A‐to‐I RNA editing in Alu sequences was significantly higher in human, which is consistent with previous studies [34, 44, 45]. The structure of the Alu repeat region sequence is a major prerequisite for most A‐to‐I RNA editing. Osenberg *et al*. [46] described A‐to‐I RNA editing and spontaneous differentiation in human embryonic stem cell (hESCs) neurons [46]. They pointed out the high level of RNA editing in Alu repeat elements in the hESCs. In our study, we also found that A‐to‐I editing events occur more frequently in Alu sequences during early embryonic development, and RNA editing is critical for the viability and normal development of organisms. In this study, we focused on RNA editing in the CDSs. The results showed that nonsynonymous editing was less frequent than synonymous editing. In addition, our results not only indicate that RNA editing was different between Ess and Noness genes but also showed that nonsynonymous editing frequency is significantly different from synonymous editing frequency. A study of the A‐to‐I editing sites by Xu and Zhang [46] showed similar results, which showed that RNA editing was less common in Ess genes than in Noness genes [47]. After removing the known SNPs, we obtained all the genes that were nonsynonymously edited. We have selected the potential genes, and in the human results, the *HOX* family genes were more novel. In the early embryonic development of pig, we found that these genes are mainly involved in fat metabolism. Moreover, Zhang *et al*. studied the reprogramming of iPSCs in pig and analyzed the expression of pig polymorphic genes [40]. The results showed that lipid metabolism contributed to the derivation of pig embryonic stem cells. Among them, *ECI1* changed the secondary and tertiary structure of the protein by RNA editing. As an important domestic animal, pig is not only the main source of meat for human beings but also an indispensable model animal in biomedical research. In the breeding process of pigs, it is very important to increase the exploration of economic traits of pigs. We performed a detailed comparative analysis of the RNA editing levels of pig embryo development to expect to find genes associated with pig economic traits, such as participation in fat metabolism. In general, we have provided new insights into the breeding of pigs.

Our study was novel because we performed RNA editing analysis on the early embryonic developmental stages of three species. The purpose of this study was to mine genes that have undergone RNA editing during early embryonic development, and to explore the distribution of RNA editing in CDSs and non‐CDSs, with particular attention to the editing sites associated with embryonic stem genes, to provide insights into the maintenance of stem cell characteristics. Our next step is to show a conservative relationship among the three species. Also, the relationship between RNA editing differences and RNA editing enzymes in early embryonic development remains to be further revealed.

## Conflict of interest

The authors declare no conflict of interest.

## Author contributions

MZL, JLH and ZHL conceived and supervised the study; TTL, QL and HL designed experiments; XX, AWD and XGW performed experiments; TTL, QL and HL analysed data; TTL, NZ and ZYC drafted the manuscript; XGW, JYH, AWD and TL made manuscript revisions. All authors read and approved the final manuscript.

## Supporting information


**Fig. S1.** Enrichment analysis of the edited genes occurring on chromosome.
**Fig. S2.** The ratio of RNA editing before and after exonerate v2.4.0 was used.
**Fig. S3.** Dynamic changes of nonsynonymous editing genes.
**Fig. S4.** Gene enrichment analysis of the intersection of nonsynonymous editing genes in three species during the development of the early embryo.
**Fig. S5.** The editing frequency of the intersection genes of human, mouse and pig in different cells.
**Fig. S6.** Tertiary structure of protein before and after RNA editing.

**Table S1.** Statistics of the samples.

**Table S2.** Numbers and percentages of RNA editing sites in the human identified by REDItools using RNA reads as inputs.

**Table S3.** Statistics for nonsynonymous and synonymous editing sites in different stage.

**Table S4.** Essential and nonessential genes in human and mouse.

**Table S5.** GO analyzed 169 genes that intersect in human, mouse and pig.

**Table S6.** GO analysis for genes that produced nonsynonymous editing.

## Data Availability

The RNA‐seq from pig has been uploaded to the GEO database (https://www.ncbi.nlm.nih.gov/geo/) under series GSE139512.
