# Supplementary material for: Pig‐specific RNA editing during early embryo development revealed by genome‐wide comparisons
Source: FEBS Open Bio. 2020 Jun 25;10(7):1389–402. doi: 10.1002/2211-5463.12900 (PMC7327910; doi:10.1002/2211-5463.12900)
Supplement: Supplementary file 1 — Fig. S1. Enrichment analysis of the edited genes occurring on chromosome. Fig. S2. The ratio of RNA editing before and after exonerate v2.4.0 was used. Fig. S3. Dynamic changes of nonsynonymous editing genes. Fig. S4. Gene enrichment analysis of the intersection of nonsynonymous editing genes in three species during the development of the early embryo. Fig. S5. The editing frequency of the intersection genes of human, mouse and pig in different cells. Fig. S6. Tertiary structure of protein before and after RNA editing. [file FEB4-10-1389-s001.docx]

**Supplementary information**


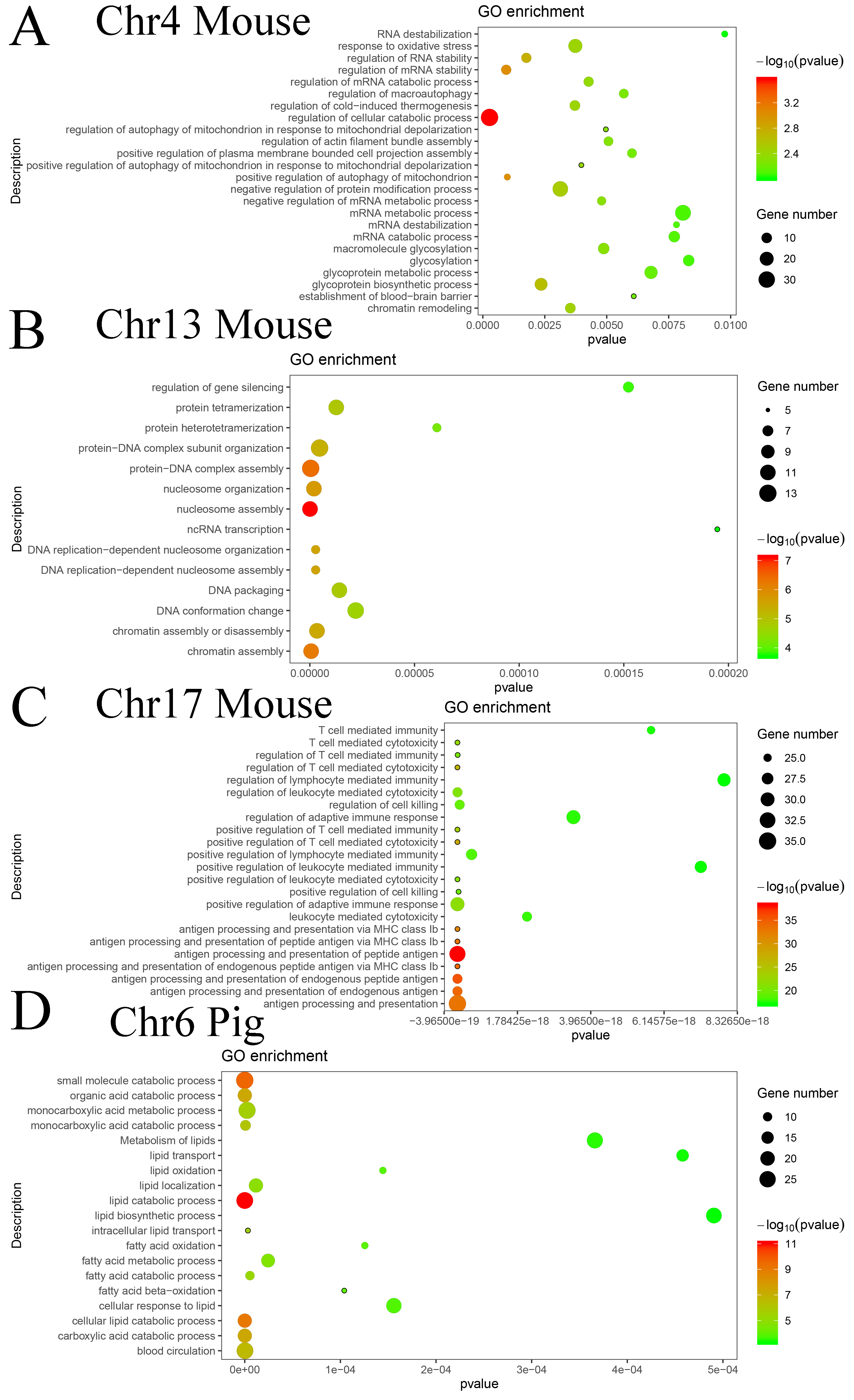


Figure S1. GO enrichment analysis of the edited genes. A. Function of edited genes occurring on chromosome 4 in mouse is related to RNA processing. B. Functions of the edited genes occurring on chromosome 13 in mouse are related to DNA replication and chromatin assembly. C. Function of the edited genes occurring on mouse chromosome 17 in mouse is related to the immune process. D. Function of the edited genes occurring on chromosome 6 in pig is mainly related to lipid metabolism.


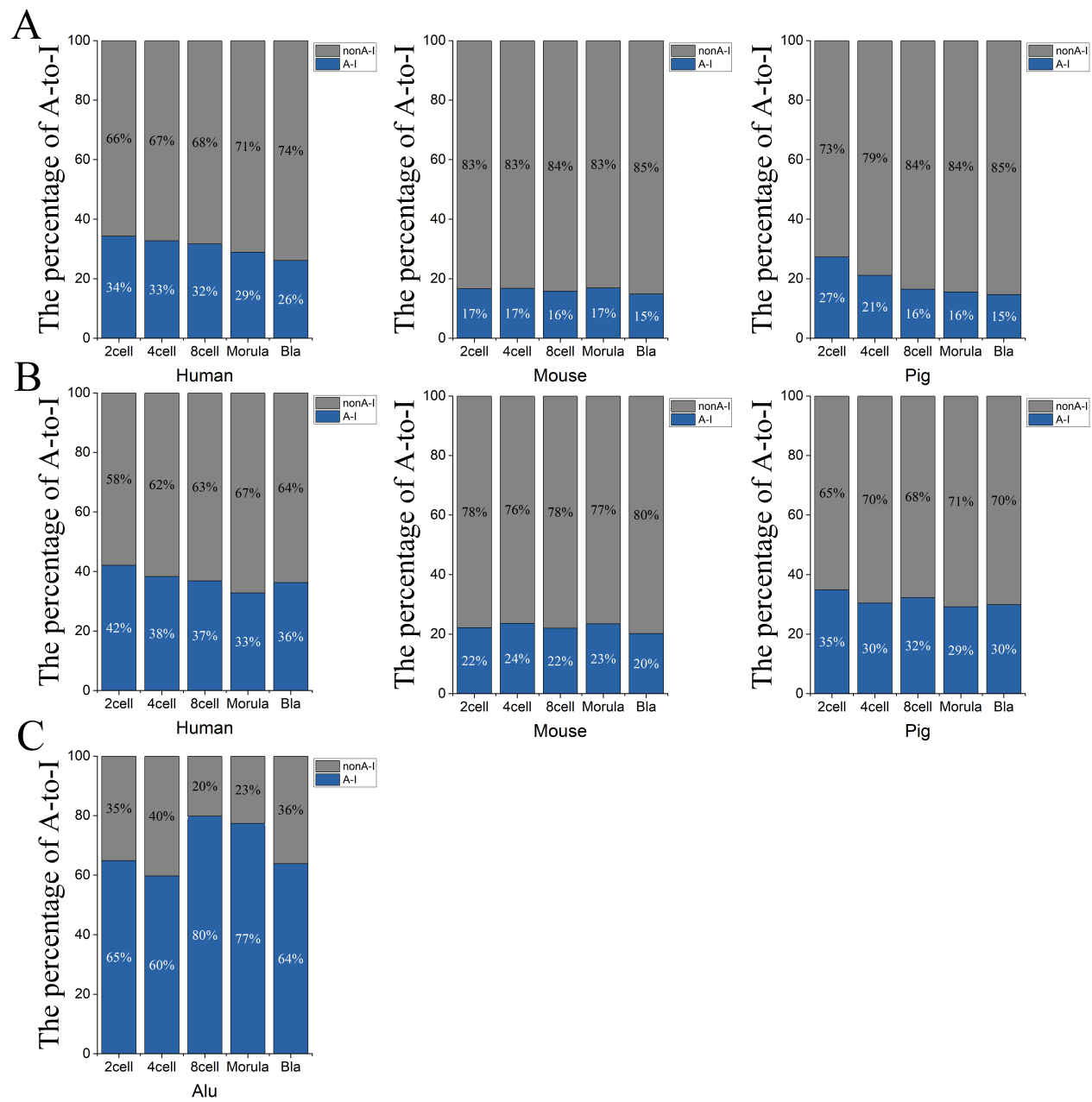


Figure S2. The ratio of RNA editing before and after exonerate v2.4.0 was used.. A. Ratio of RNA editing prior to treatment with exonerate v2.4.0. B. The ratio of RNA editing after exonerate v2.4.0 was used. C. The proportion of A-I in human Alu is significantly higher.


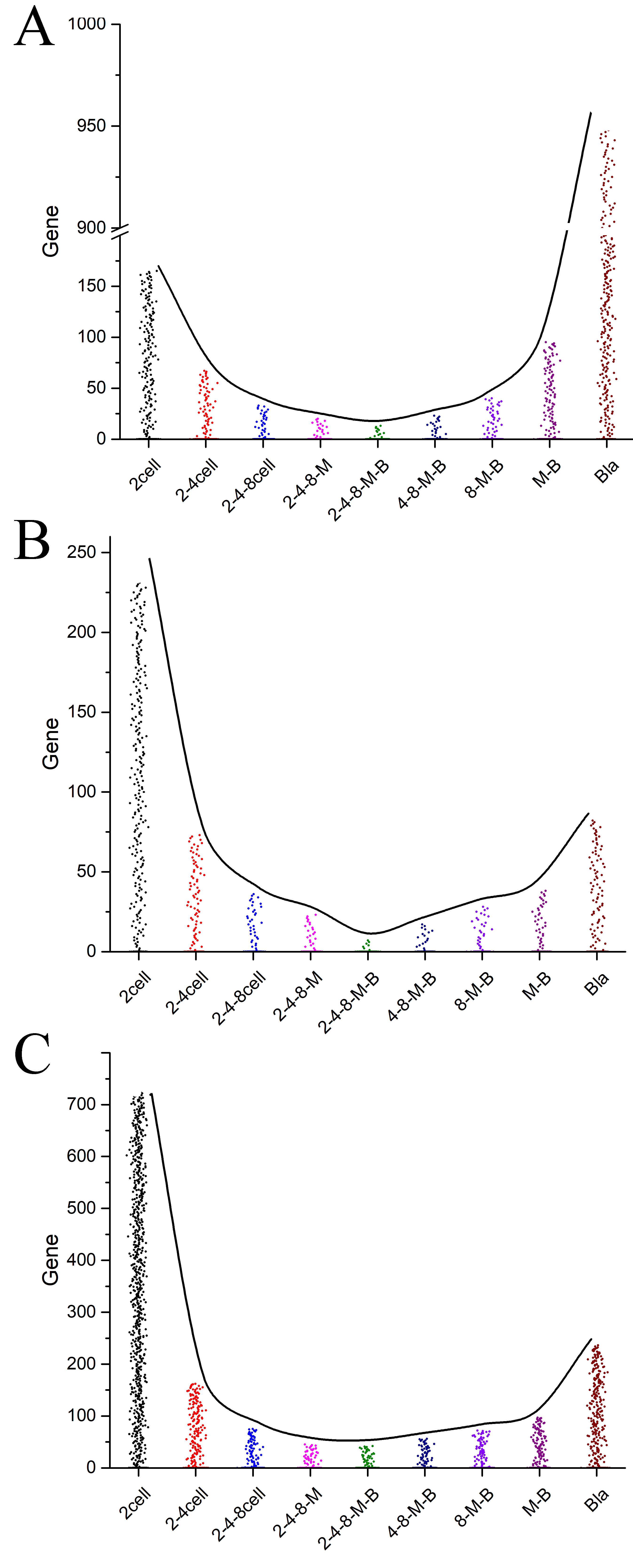


Figure S3. Dynamic changes of non-synonymous editing genes. (ABC)From top to bottom, the dynamic changes of non-synonymous editing genes in human, mouse and pig during early embryo development were in turn. A point represents a gene.


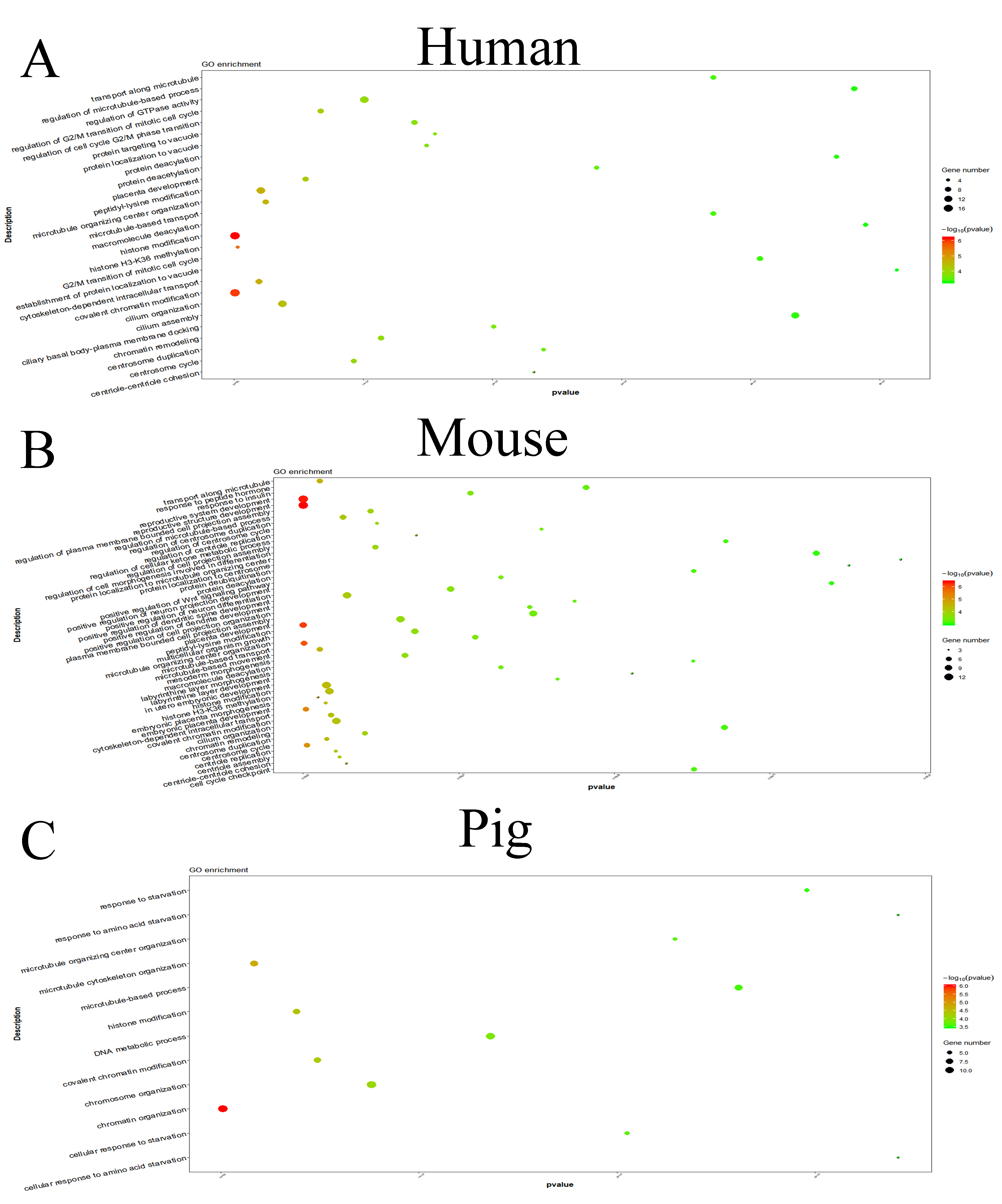


Figure S4. Gene enrichment analysis of the intersection of non-synonymous editing genes in three species during the development of the early embryo. From top to bottom species are human, mouse and pig.


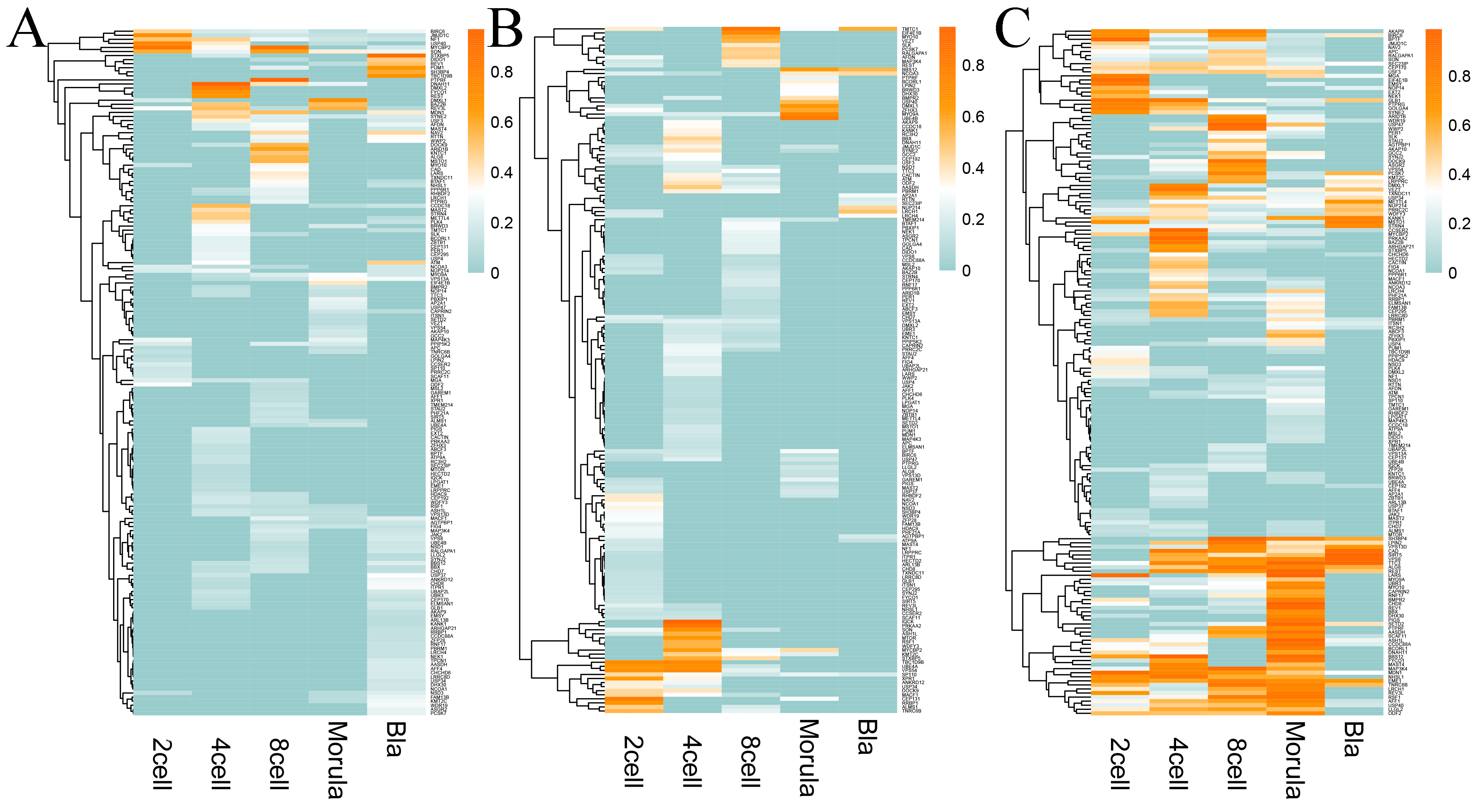


Figure S5. The editing frequency of the intersection genes of human, mouse and pig in different cells. Here are the 169 genes shared by humans, mouse and pig. From left to right species are human, mouse and pig.


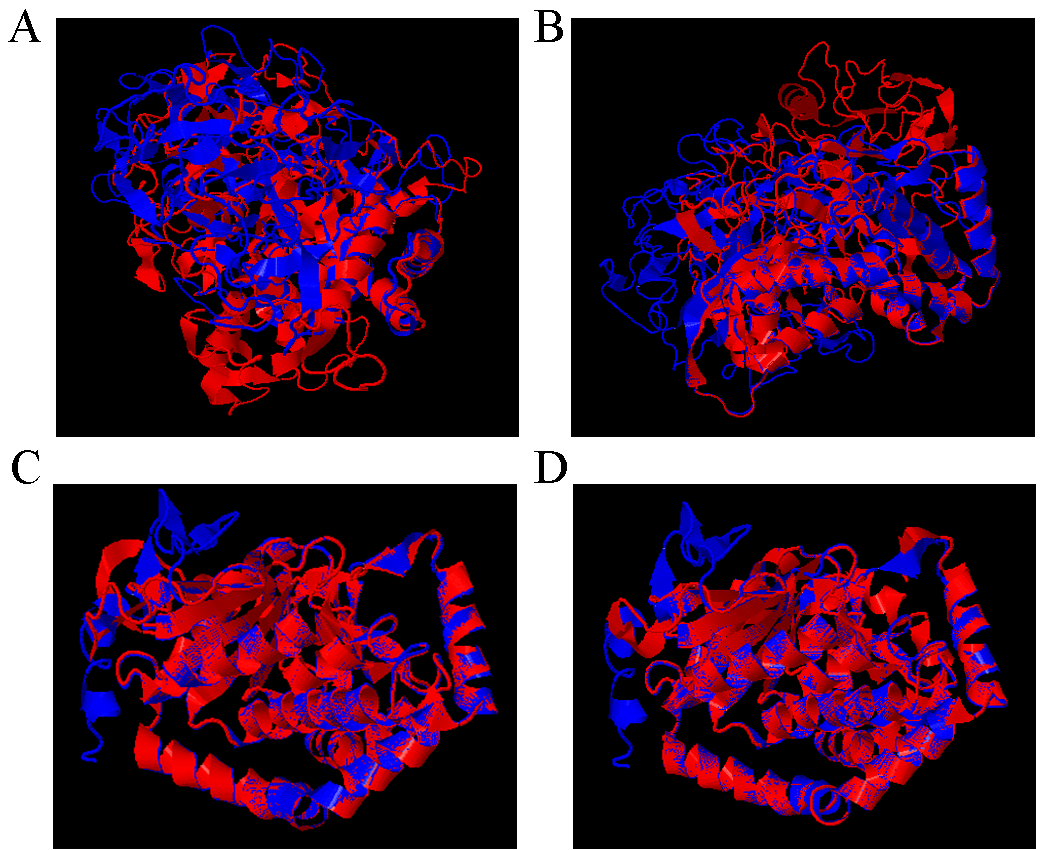


Figure S6. Tertiary structure of protein before and after RNA editing. (AB)Tm-align was used to compare the structure before and after amino acid changes at positions 401 and 402. (CD)Tm-align was used to compare the structure before and after amino acid changes at positions 90 and 17 in ECI1. This showed the superposed full-atom structure of the entire chain. Blue: the structure before RNA editing occurs; Red: the structure after RNA editing.
